# Genomic scan for quantitative trait loci of chemical and physical body composition and deposition on pig chromosome X including the pseudoautosomal region of males

**DOI:** 10.1186/1297-9686-41-27

**Published:** 2009-03-11

**Authors:** Carol-Anne Duthie, Geoff Simm, Miguel Pérez-Enciso, Andrea Doeschl-Wilson, Ernst Kalm, Pieter W Knap, Rainer Roehe

**Affiliations:** 1 Animal Breeding and Development, Sustainable Livestock Systems Group, Scottish Agricultural College, West Mains Road, Edinburgh, EH9 3JG, UK; 2 ICREA, Dept. Food and Animal Science, Universitat Autònoma de Barcelona, 08193 Bellaterra, Spain; 3 Institute of Animal Breeding and Husbandry, Christian-Albrechts-University of Kiel, Hermann-Rodewald-Strasse 6, D-24118 Kiel, Germany; 4 PIC Germany, Ratsteich 31, D-24837 Schleswig, Germany

## Abstract

A QTL analysis of pig chromosome X (SSCX) was carried out using an approach that accurately takes into account the specific features of sex chromosomes *i.e*. their heterogeneity, the presence of a pseudoautosomal region and the dosage compensation phenomenon. A three-generation full-sib population of 386 animals was created by crossing Pietrain sires with a crossbred dam line. Phenotypic data on 72 traits were recorded for at least 292 and up to 315 F_2 _animals including chemical body composition measured on live animals at five target weights ranging from 30 to 140 kg, daily gain and feed intake measured throughout growth, and carcass characteristics obtained at slaughter weight (140 kg). Several significant and suggestive QTL were detected on pig chromosome X: (1) in the pseudoautosomal region of SSCX, a QTL for entire loin weight, which showed paternal imprinting, (2) closely linked to marker SW2456, a suggestive QTL for feed intake at which Pietrain alleles were found to be associated with higher feed intake, which is unexpected for a breed known for its low feed intake capacity, (3) at the telomeric end of the q arm of SSCX, QTL for jowl weight and lipid accretion and (4) suggestive QTL for chemical body composition at 30 kg. These results indicate that SSCX is important for physical and chemical body composition and accretion as well as feed intake regulation.

## Introduction

To understand the genetic control of economically important traits in pigs a large number of studies have investigated QTL that contribute to variation in these traits *e.g*. [1-4]. Most QTL have been identified on autosomes with only a few on the sex chromosomes. One reason may be that the role of sex chromosomes in the genomic regulation of these traits is less important. Another reason may come from the fact that, until recently, software modelling more appropriately the specific features of sex chromosomes was not available. Indeed, the mammalian X chromosome is considerably larger than the Y chromosome and carries more genes [5,6]. For example, 1 250 genes are known on the human X chromosome but only 147 on the Y chromosome [7]. As a result, female cells, which carry two copies of the X chromosome, contain twice as many X-linked genes than males. Mammals have developed a mechanism to balance the dosage of the X chromosome genes between sexes, called the dosage compensation phenomenon [8,9]. Furthermore, chromosomes X and Y only share a small homologous region called the pseudoautosomal region [10].

Because QTL mapping software taking into account the specific X chromosome features was not available, most studies have adopted a regression based approach analyzing males and females separately, which decreases the power of QTL detection *e.g*. [11,1,12]. Recently, Perez-Enciso *et al*. [13] have developed software combining a mixed model methodology and a maximum likelihood approach, which can model the specific X chromosome features in a QTL analysis.

Therefore, the aim of the present study was to investigate QTL on pig chromosome X (SSCX) for chemical and physical body composition and deposition using a methodology, which accurately takes into account the features associated with this chromosome.

## Methods

### Animal resources

This study was based on data recorded from a three-generation full-sib design, developed from a cross between seven unrelated Pietrain grandsires, all heterozygous (Nn) at the *ryanodine receptor 1 (RYR1) *locus, and 16 unrelated grand-dams from a three-way cross of Leicoma boars with Landrace x Large White dams. Eight boars from the F_1 _generation, were mated to 40 sows over two parities to produce the F_2 _generation comprising 315 pigs. Forty-eight gilts and 46 barrows of the F_2 _generation were housed individually in straw-bedded pens and fed manually with a weekly recording of feed consumption. The remaining animals (117 gilts and 104 barrows) were housed in mixed sex groups of up to 15 pigs in straw-bedded pens. Animals housed in groups were fed with an electronic feeding station (ACEMA 48), which recorded feed consumption at each visit. According to body weight ranges, three different pelleted diets were provided *i.e*. containing 13.8 MJ ME/kg and 1.2% lysine for range 30–60 kg, 13.8 MJ ME/kg and 1.1% lysine for 60–90 kg and 13.4 MJ ME/kg and 1.0% lysine for 90–140 kg. Maximal protein deposition was reached by providing pigs with *ad libitum *access to diets, which were formulated slightly above requirement. For a more detailed description of the project management see Landgraf *et al*. [14,15] and Mohrmann *et al*. [16,17].

### Physical body composition

Pigs were slaughtered at 140 kg body weight in a commercial abattoir. Phenotypic measurements on 37 traits related to physical body composition were collected by two methods, the AutoFOM device and dissection. The AutoFOM device used an automatic ultrasound scanning technique to produce a three-dimensional image of the pig [18], which provided measurements of valuable carcass cuts, including average fat thickness, belly weight, lean content, lean content of the belly and weights of entire and trimmed shoulder, loin and ham without bones. The right side of each carcass was dissected into weights of the primal cuts, neck, shoulder, loin, ham and belly. The former four cuts were dissected into lean and fat tissue. Furthermore, weights of the jowl, thick rib, flank, front as well as hind hock, tail and claw were recorded. Further measurements were made on the cold left carcass side, for carcass length, sidefat thickness, fat content and area of the belly as well as loin eye area, fat area, and thinnest fat measure (fat degree B) calculated at the 13^th^/14^th ^rib interface. Landgraf *et al*. [14] have described more precisely the dissection of carcasses.

### Chemical body composition

Phenotypic information was obtained for 25 traits related to chemical body composition and deposition. Protein content of the loin and intramuscular fat content were measured by near-infrared reflectance spectroscopy in the *musculus longissimus thoracis et lumborum*. The deuterium dilution technique, an *in vivo *method determining chemical body composition based on body water was used to determine protein, lipid and ash contents of the empty body at target body weights of 30, 60, 90, 120, and 140 kg. The accuracy of this technique has been verified in previous studies using magnetic resonance imaging on live animals [17] and chemical analysis of serially-slaughtered animals [15]. This method measures the water content of the empty body, from which the percentage of fat-free substance of the empty body can be estimated. Based on the percentage of the fat-free substance, protein and ash content of the empty body are estimated, and lipid content is the deviation of the fat-free content from one. The equations for estimating these chemical components were developed by Landgraf *et al*. [15] using the data of the F_1 _generation of the three-generation full-sib population analyzed in the present study. Protein and lipid accretion rates at four stages of growth were calculated as the difference between protein or lipid composition at two consecutive target weights divided by days of growth between the target weights. Furthermore, daily gain, feed intake and food conversion ratio were recorded at different stages of growth. Means and standard deviations of the 72 traits analyzed in the present study are presented in Additional file 1, Table S1.

### Genotypic data

Blood samples were collected from F_0_, F_1 _and F_2 _animals from the *vena jugularis *and DNA was isolated. All animals were genotyped for eight informative microsatellite markers covering SSCX. Markers and their distances were taken from the published USDA linkage map [19], which provided all information on their positions and alleles (Table 1). The average distance between markers was 18.3 cM and the largest gap was 22.4 cM. Linkage analysis was performed with Crimap [20]. The marker order agrees with the USDA linkage map but distances between markers differ from those in the USDA linkage map, which is probably due to the fact that the marker coverage in the present study is not as good as in the USDA map.

**Table 1:** Markers used in the present QTL mapping project, their relative map position based on the USDA pig map, their position from linkage analysis using CRIMAP in this experimental population, number of different alleles, heterozygosity in F_1 _generation (H) and polymorphic information content in the F_2 _generation (PIC)

**Marker**	**Position (cM)** **USDA MAP**	**Position (cM) experimental data**	**Number of alleles**	**H**	**PIC**
*SW949*	0.0	0.0	6	0.65	0.53
*SW980*	11.9	36.2	7	0.87	0.80
*SW1903*	33.0	46.4	5	0.87	0.70
*SW2456*	55.4	56.5	6	0.81	0.67
*SW259*	74.4	63.6	5	0.89	0.70
*SW1943*	87.4	73.5	5	0.70	0.70
*SW707*	107.9	78.2	4	0.49	0.59
*SW2588*	128.4	79.8	4	0.25	0.37

### Statistical analysis

QTL mapping was carried out using the software QxPak version 2.16 [13]. This program uses mixed models and the maximum likelihood method to estimate the QTL location and effects. A fixed effects model that estimates additive and dominance effects was chosen for the QTL analysis. In cases where the dominance effect was not significant, the analysis was repeated with an additive-only model. Maternal and paternal imprinting was tested for only in the pseudoautosomal region. In this analysis, the additive estimate is defined as half of the difference between animals homozygous for alleles from the grand-paternal sire line and those homozygous for alleles from the grand-maternal dam line. A positive additive genetic value indicates that the allele originating from the grand-paternal sire line (Pietrain) showed an increasing QTL effect compared to the allele from the grand-maternal dam line and *vice versa*. The dominance effect is defined as the deviation of heterozygous animals from the mean of both types of homozygous animals. Fixed effects and covariates were fitted in the models depending on their significance for the trait. Sex, ryanodine receptor genotype (MHS-genotype) and batch were included in the model for all traits. In addition, housing was included as a fixed effect for feed intake and food conversion ratio traits. Body weight at slaughter was fitted in the model as a covariate for carcass characteristics measured at slaughter. For chemical body composition traits measured at different target weights, body weight at that target weight was fitted in the model as a covariate. Protein and lipid accretion, daily gain, feed intake and food conversion ratio were adjusted for the small differences between target and actual body weight at the start and end of the considered weight range. The analysis provides likelihood ratios under the models tested and associated nominal P-values. A previous study by Perez-Enciso *et al*. [21] has shown that nominal P-values 0.005 and 0.001 correspond to 5% and 1% chromosome-wide significant P-values, respectively, based on the chi-squared distribution with two degrees of freedom. Therefore, in the present study, nominal P-values <0.001, 0.005 and 0.01 were treated as significant at the 1%, 5% and suggestive at the 10% chromosome-wide level, respectively. Confidence intervals of QTL were estimated using the 1-LOD drop method [22].

### Methodology

The mixed model methodology applied in this study includes all pedigree information and uses the Maximum Likelihood Method to estimate the QTL effects. Due to the flexibility of the methodology, we were able to take into account the heterogeneity between the sex chromosomes, the presence of the pseudoautosomal region and the sex chromosome dosage compensation phenomenon. The methodology applied here is described in Perez-Enciso *et al*. [23] and implemented in the programme QxPak.

In more detail, the main issues relate to modelling the mammalian dosage compensation and computing the *p*_*s*_, *ρ*_*sA *_and *ρ*_*sB *_coefficients. *p*_*si *_is the average probability for the *i*th individual of a gene within segment *s *to originate from breed A. *ρ*_*sA*(*i*, *i*')_, *ρ*_*sB*(*i*, *i*') _is the probability of individuals *i *and *i' *having received identical-by-descent (IBD) alleles of breed A(B).

In the non-pseudoautosomal region of the X chromosome the male phenotype is expressed as:

(1) *γ*_*M *_= *μ*_*M *_+ *g*^2 ^+ *e*,

and the female phenotype as:

(2) *γ*_*F *_= *μ*_*F *_+ *ψ*^1^*g*^1 ^+ *ψ*^2^*g*^2 ^+ *d*_*g*1, *g*2 _+ *e*,

*μ *is sex mean; g^*i*^, the genetic origin, indicates the haplotype origin, 1 for male and 2 for female; *ψ*^*h *^is the dosage compensation effect for *h*th haplotype allele effect and *d *is the dominance interaction. In this case, interaction between alleles (dominance) can be estimated only in females. The allele contributing to the male phenotype always comes from the mother (*g*^2^). Parameters *ψ*^1 ^and *ψ*^2 ^should always add up to 1.

The genetic covariances between two crossed individuals are calculated as:

if *i *and *i' *are both males

(3) $$Cov(g_{i},g_{i'})=\mathrm{Pr}(g_{i}^{2}\equiv g_{i'}^{2}\in A)\sigma_{Ag}^{2}+\mathrm{Pr}(g_{i}^{2}\equiv g_{i'}^{2}\in B)\sigma_{Bg},$$

if *i *is a male and *i' *is a female

(4) $$Cov(g_{i},g_{i'})=\sum_{h=1}^{2}\psi^{h}[\mathrm{Pr}(g_{i}^{2}\equiv g_{i'}^{h}\in A)\sigma_{Ag}^{2}+\mathrm{Pr}(g_{i}^{2}\equiv g_{i}^{h}\in B)\sigma_{Bg}^{2}],$$

if *i *and *i' *are both females

(5) $$Cov(g_{i,}g_{i'})=\sum_{h=1}^{2}\sum_{h'=1}^{2}\psi^{h}\psi^{h'}[\mathrm{Pr}g_{i}^{h}\equiv g_{i}^{h'}\in A)\sigma_{Ag}^{2}+\mathrm{Pr}(g_{i}^{h}\equiv g_{i'}^{h'}\in B)\sigma_{Bg}^{2}],$$

Where, $\mathrm{Pr}(g_{i}^{h}\equiv g_{i'}^{h'}\in A)$ is the probability of alleles $g_{i}^{h}$ and $g_{i'}^{h'}$ being IBD and being of breed origin A, and $\mathrm{Pr}(g_{i}^{h}\equiv g_{i'}^{h'}\in B)$ is the probability of alleles $g_{i}^{h}$ and $g_{i'}^{h'}$ being IBD and being of breed origin B. $\sigma_{Ag}^{2}$ is the variance of the gene effects in breed A and $\left(\sigma_{Bg}^{2}\right)$ is the variance of the gene effects in breed B. We define $p_{gi}=\mathrm{Pr}(g_{i}^{2}\in A)$ when *i *is a male and $p_{gi}=\sum_{h=1}^{2}\psi^{h}\mathrm{Pr}(g_{i}^{h}\in A)$ a female.

In addition, the pseudoautosomal region of the X and Y chromosomes has been considered. This is the homologous region between the X and Y chromosomes, thus in males, this is the only X chromosome region where recombination can occur [23].

## Results

The genomic analysis identified on the pig X chromosome three significant QTL and five suggestive QTL for carcass cuts, lean tissue characteristics, chemical body composition and deposition as well as daily feed intake. The additive and dominance effects of these QTL are presented in Table 2. Two QTL were identified with imprinting effects in the pseudoautosomal region, which are shown in Table 3. The correlations between these traits are presented in Additional file 2, Table S2.

**Table 2:** Evidence for quantitative trait loci (QTL) for carcass cuts, chemical body composition, lipid accretion and feed intake on pig chromosome X

**Trait^1^**	**LR^2^**	**Pos (CI)^3^**	**% Var^4^**	**a ± SE^5^**	**d ± SE^5^**
*Carcass characteristics – dissected carcass cuts*					

Entire loin weight (g)	11.56**	7 (0–27)	3.7	**283.8 ± 82.7**	-
Loin weight without external fat (g)	7.68^a^	11 (0–33)	2.5	**184.5 ± 66.1**	-
Jowl weight (g)	17.91**	128.4 (106–128.4)	5.8	57.4 ± 37.6	**216.9 ± 74.3**

*Chemical body composition and accretion rates*					

Lipid cont. empty body, 30 kg (%)	10.38^a^	83 (68–108)	3.3	-0.496 ± 0.259	**1.705 ± 0.532**
Protein cont. empty body, 30 kg (%)	10.43^a^	83 (68–109)	3.8	0.011 ± 0.006	**-0.038 ± 0.012**
Protein cont. FFS_EB_, 30 kg (%)	9.36^a^	82 (59–128.4)	3.1	**-0.093 ± 0.046**	**0.285 ± 0.095**
LAR 90–120 g (g/day)	9.60*	128.4 (78–128.4)	3.2	**26.7 ± 8.5**	-

*Daily gain, feed intake and food conversion ratio*					

DFI 120–140 g (g/day)	7.00^a^	56 (36–83)	2.3	**101.6 ± 38.2**	-

^1^Traits of FFS_EB_, LAR and DFI denote fat-free substance of the empty body, lipid accretion rate, daily feed intake, respectively

^2^LR represents likelihood ratio values and superscript ^a^implies a suggestive QTL at the 10% chromosome-wide level, whereas * and ** imply significant QTL at the 5% or 1% chromosome-wide levels, respectively

^3^Positions of the QTL in cM based on the USDA reference map and confidence intervals (CI) in brackets

^4^Percentages of F_2 _variance explained by the QTL calculated as the proportion of residual variances due to the QTL effect on the residual variances excluding the QTL effect

^5^Estimated additive (a) and dominance (d) effects and their standard errors (SE); estimates in bold represent significant additive or dominance effects

**Table 3:** Evidence for quantitative trait loci (QTL) associated with imprinting effects in the pseudoautosomal region

**Trait**	**LR^1^**	**Pos (CI)^2^**	**% Var^3^**	**a ± SE^4^**	**Imprinting**
*Carcass characteristics (lean and fat)*					

Entire loin weight (g)	10.07*	6 (0–28)	3.2	**133.3 ± 41.6**	Paternal
Neck weight without external fat^5 ^(g)	8.85*	1 (0–13)	2.8	**374.0 ± 124.8**	Maternal

^1^LR represents likelihood ratio values and * implies a significant QTL at the 5% chromosome-wide level

^2^Positions of the QTL in cM based on the USDA reference map and confidence intervals (CI) in brackets

^3^Percentages of F_2 _variance explained by the QTL calculated as the proportion of residual variances due to the QTL effect on the residual variances excluding the QTL effect

^4^Estimated additive (a) effects and their standard errors (SE); estimates in bold represent significant additive effects

^5^New QTL only identified when the imprinting effect is included in the model

### Carcass characteristics

**Figure 1:**
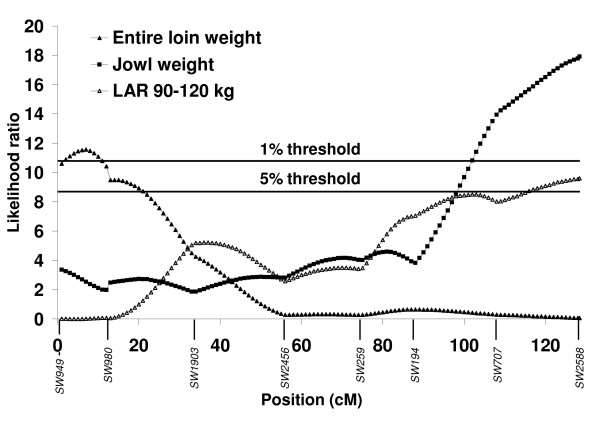
**Likelihood ratio curves for evidence of significant quantitative trait loci for entire loin weight, jowl weight and lipid accretion rate (LAR) 90–120 kg on SSCX**. Positions in cM are based on the USDA reference map.

**Figure 2:**
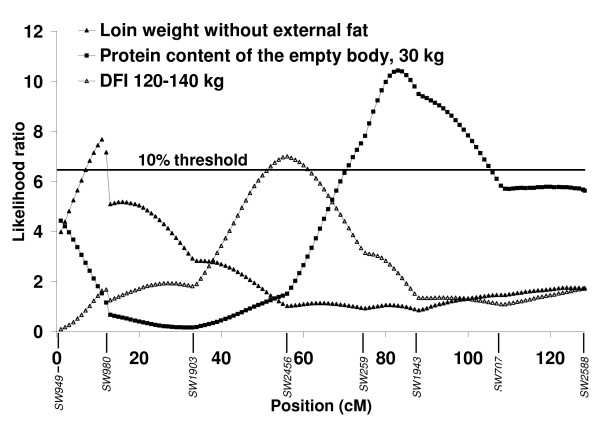
**Likelihood ratio curves for evidence of suggestive quantitative trait loci for loin weight without external fat, protein content of the empty body at 30 kg body weight and daily feed intake (DFI) 120–140 kg on SSCX**. Positions in cM are based on the USDA reference map.

A QTL significant at the 1% chromosome-wide level was identified for entire loin weight in the pseudoautosomal region of SSCX at 7 cM between *SW949 *and *SW980 *explaining 3.7% of the phenotypic variance (Figure 1). The significant additive effect at this QTL indicates that the grand-paternal Pietrain breed is associated with a 284 g higher loin weight. At a similar location in the pseudoautosomal region *i.e*. at 11 cM, a suggestive QTL was identified for loin weight without external fat explaining 2.5% of the phenotypic variance (Figure 2). The significant additive effect at this QTL indicates that the grand-paternal Pietrain breed is associated with a 185 g higher lean meat weight of the loin cut. At the telomeric end of the q arm of SSCX, at the same position as *SW2588 *(128.4 cM) a QTL significant at the 1% chromosome-wide level was identified for jowl weight accounting for 5.8% of the phenotypic variance (Figure 1). The significant dominance effect at this QTL indicates that heterozygous animals are associated with a 217 g higher jowl weight.

### Chemical body composition and accretion

At the telomeric end of the q arm of SSCX, at the same position as the QTL for jowl weight and *SW2588 *(128.4 cM), a QTL significant at the 5% chromosome-wide level was identified for lipid accretion rate during the growth period between 90 and 120 kg (Figure 1). This QTL accounts for 3.2% of the phenotypic variance and the significant additive effect indicates that the purebred Pietrain breed is associated with a 27 g higher lipid accretion rate at this growth period. Suggestive QTL for protein content of the fat-free substance and protein and lipid content of the empty body were identified between *SW259 *and *SW1943 *at 82–83 cM explaining 3.1, 3.8 and 3.3% of the phenotypic variance, respectively (Figure 2). These traits showed similar likelihood ratio profiles as they are closely correlated (Additional file 2, Table S2). Pietrain alleles associated with decreased additive genetic effects of protein content of the fat-free substance are found at these QTL and heterozygous animals showed dominance effects associated with increased lipid content of the empty body, decreased protein content of the empty body and increased protein content of the fat-free substance at 30 kg body weight.

### Daily gain, feed intake and food conversion ratio

A single suggestive QTL was identified for daily feed intake at a late stage of growth (120–140 kg) in a region of SSCX (56 cM) where no other QTL were identified (Figure 2). This QTL accounts for 2.3% of the phenotypic variance and the significant additive effect indicates that Pietrain alleles are associated with a 102 g/day higher feed intake at this stage of growth.

### Imprinting in the pseudoautosomal region

Two QTL with significant imprinting effects were identified in the pseudoautosomal region (Table 3). At 6 cM, significant paternal imprinting effects were identified for entire loin weight, indicating that only the maternal allele is expressed at this QTL. A QTL with significant maternal imprinting effects was identified at 1 cM for neck weight without external fat, indicating that only the paternal allele is expressed at this QTL. This QTL for neck weight without external fat was identified only when imprinting was considered in the analysis.

## Discussion

The aim of the present study was to investigate QTL on pig chromosome X for traits of carcass characteristics, chemical and physical body composition and accretion rates as well as daily gain, feed intake and food conversion ratio considering the specific features of the sex chromosomes as described in the introduction. There is evidence in the literature for QTL on pig chromosome X involved in carcass characteristics, lean tissue, growth and fatness *e.g*. [24,2,26,4]. In particular, the study by Milan *et al*. [2] reported QTL on SSCX with the largest effects for leanness and fatness traits in a cross between the French Large White and the Meishan breeds. In the present study, QTL were identified on SSCX for carcass characteristics (entire carcass cuts and lean tissue), chemical body composition, lipid accretion as well as feed intake. The QTL analysis is based on animals from an F_2 _full-sib design of crosses between Pietrain boars and crossbred commercial dams in order to reflect the commercial product of growing-finishing pigs. Therefore, the QTL alleles in the dam founder may not be fixed, which has to be considered in the interpretation of the results.

In pigs, the pseudoautosomal region lies at the telomeric end of the p arm of SSCX and covers a ~11 cM region homologous to the Y chromosome. In the present study, this region showed important associations with entire loin weight and lean meat of the loin cut (Figure 1 and Figure 2). The purebred Pietrain breed is associated with higher loin weight (284 g) and lean meat weight of the loin cut (185 g). Within the pseudoautosomal region of SSCX, QTL for entire carcass cuts and lean tissue have also been reported in the literature [24,1,26]. A previous genomic analysis on the autosomes using the same phenotypic data as the present study [27,16], detected Pietrain alleles for QTL on SSC2, SSC6, SSC8, SSC9 and SSC13 also associated with increased weights of carcass cuts and lean tissue. However, this was not the case on SSC14 on which a Pietrain allele was associated with decreased weights of these characteristics, suggesting a cryptic gene in a breed selected over a long period for leanness. Although pseudoautosomal regions exist in other mammals, their length and gene content seem to be variable, for example mouse and human pseudoautosomal regions are totally non-homologous. Previously, this region was considered to have an important role in meiotic pairing and male fertility. However the variability in gene content of this region across species and the absence of this region in marsupials, suggest that it may not be so important for fertility in mammals [10]. The results of the present study indicate that this region in pigs contains genes, which influence carcass characteristics.

QTL for lean tissue characteristics *e.g*. [1,2,4] have been detected in regions other than the pseudoautosomal region of SSCX. In the present study no QTL for lean tissue was identified, which suggests that the favorable alleles for lean tissue may already be fixed in the populations analyzed here. Another surprising result is that no QTL for fatness was identified although many reports have described fatness QTL on pig SSCX *e.g*. [25,2,26,3,28]. Most of these studies were based on crosses between breeds characterized by a high leanness and breeds characterized by a high fatness *i.e*. Meishan, wild Boar or Iberian breeds. Therefore, these QTL may not be segregating in our population, which has been selected for leanness over a long time.

It is likely that the genomic regulation of chemical body components and their accretion is a complex process involving more than one genomic region and regulated differently throughout growth. Measurements of chemical body composition in live animals are expensive. Therefore, studies on QTL associated with these traits are limited to two studies analyzing the data of the present population across several autosomes [27,16]. In the present study, we have identified a significant QTL for lipid accretion rate at 90–120 kg on SSCX, while a previous report by Duthie *et al*. [27] detected QTL for the same trait on two autosomes at 60–90 kg on SSC8 and at 120–140 kg on SSC9. Pietrain alleles were found to be associated with increased lipid accretion rate at 60–90 kg on SSC8 and 90–120 kg on SSCX. A significant dominance effect was identified at the QTL for lipid accretion rate 120–140 kg on SSC9, however no dominance effect was identified on SSCX. The QTL for lipid accretion rate identified on SSCX is in a region containing no QTL for fat tissue, unlike the QTL detected on SSC8 and 9, which were close to numerous QTL for subcutaneous fat (SSC9) and a QTL for intramuscular fat (SSC8) [27]. This result is surprising because many QTL for fat tissue traits have been reported around this SSCX region [2,23,28]. In the same location as the SSCX QTL associated with lipid accretion rate, a QTL for jowl weight was found (Figure 1). Heterozygous animals had a higher weight of the jowl cut. A previous analysis of the same phenotypic data also revealed a QTL for jowl weight on SSC1 [16] but heterozygous animals at this QTL had a lower jowl weight. Furthermore, a significant additive effect indicated that Pietrain alleles were associated with lower jowl weight on SSC1. To our knowledge, there is no other data in the literature for such QTL.

Suggestive QTL were identified in the present study for chemical body composition at an early growth stage (30 kg body weight), in a region of SSCX where no other QTL were identified (Figure 2). Previously, QTL for chemical body composition for early growth stages have also been identified on autosomes: 30 kg, SSC6, 60 kg, SSC6 and SSC9 [27,16]. At the QTL on SSC6 and SSC9 for chemical body composition at 30 kg and 60 kg, respectively, significant dominance effects indicate that heterozygous animals are associated with decreased protein content of the fat-free substance and lipid content of the empty body, but increased protein content of the empty body. In contrast, at the QTL on SSCX and SSC6 for chemical body composition at 30 kg and 60 kg respectively, significant dominance effects indicate that heterozygous animals are associated with increased protein content of the fat-free substance and lipid content of the empty body, and decreased protein content of the empty body. The QTL likelihood ratio profile for protein content of the empty body is almost identical to the QTL for lipid content of the empty body, which is expected for traits that change proportionally in opposite directions. A large number of QTL have been reported for lean and fat tissue as well as for growth *e.g*. [29,24,2,28] in the same SSCX region as that containing QTL for chemical body composition. Therefore it is surprising that no QTL were identified in this study for physical body composition traits in this region of SSCX.

Cepica *et al*. [30] have assigned seven genes between markers *SW259 *and *SW1943*, within the same region as the QTL for chemical body composition identified in the present study. Based on location and function, *Acyl-CoA synthetase long-chain 4 *gene (*ACSL4*) is a potential positional candidate gene for the QTL for chemical body composition in the present study because it plays a key role in the metabolism of fatty acids and thus energy balance [31].

A suggestive QTL for daily feed intake for the growth stage 120–140 kg was identified in a region of SSCX where no other QTL were identified in the present study (Figure 2). Our previous analysis with the same population data on several autosomes, identified significant QTL for daily feed intake for growth periods 60–90 kg on SSC6 and SSC10, 90–120 kg on SSC6 and 120–140 kg on SSC2 [27,16]. Pietrain alleles were associated with decreased feed intake at 60–90 kg (SSC10), as expected for a breed, which has been intensively selected for lean content [32,33]. However, the Pietrain alleles (cryptic) at the SSCX QTL are associated with a 102 g higher feed intake at 120–140 kg. This is unexpected, as the Pietrain breed is well known for its low feed intake capacity. Within the same marker bracket (*SW2456-SW259*), Cepica *et al*. [24] have reported a QTL for food consumption in a population derived from a cross between wild Boar and Meishan.

Imprinting can be analyzed only in the pseudoautosomal region of the X chromosome, where X and Y chromosomes are homologous. Imprinting analysis is important to achieve a better understanding of the genetic control of important traits and to uncover QTL, which cannot be detected from an analysis considering only additive and dominance effects. In the present study, the QTL for entire loin weight showed paternal imprinting indicating that only the maternal allele is expressed at this QTL. Moreover, a QTL for neck weight without external fat was identified, which was not detected in the analysis without imprinting modelling. At this QTL, maternal imprinting was identified indicating that only the paternal allele is expressed. To the best of our knowledge, there is no information in the literature, reporting imprinting within the pseudoautosomal region of SSCX.

The literature is sparse for QTL on chromosome X in other livestock species. In cattle and sheep, no QTL have been reported for similar traits on the X chromosome. In cattle, only four QTL have been reported on the X chromosome for reproduction and disease resistance traits [34,35] and in sheep a single QTL has been reported for parasite resistance [36]. There is limited evidence for QTL affecting production traits in these species and more effort is needed to detect such QTL. Unlike in mammals, in chickens sex determination operates through a ZZ/ZW sex chromosome system in which females represent the heterogametic sex (ZW) and males the homogametic sex (ZZ) [37]. A large number of QTL for production traits have been identified on the Z sex chromosome *e.g*. [38-41].

## Conclusion

The results of the present study indicate that pig chromosome X is involved in the genomic regulation of physical and chemical body composition as well as growth and feed intake. Our previous analysis of the same data set over several autosomes [27,16] detected a larger number of significant QTL, indicating that the role of SSCX is probably less important in the regulation of economically important traits in pig production than that of autosomes. However, the QTL found on SSCX did account for similar proportions of the phenotypic variance. In summary, the present results on sex-linked QTL in pigs give more insight into the sex related genomic regulation of these traits, which may be based on different features from those on autosomal chromosomes.

## Competing interests

The authors declare that they have no competing interests.

## Authors' contributions

CAD performed the data analysis, wrote and prepared the manuscript for submission. RR was the principle supervisor of the study and assisted with preparation of the manuscript. GS, ADW, EK and PWK co-supervised the study and reviewed the manuscript. MPE provided assistance with the data analysis and reviewed the manuscript. All authors read and approved the final manuscript.

## Supplementary Material

Additional File 1 **Table S1.** Means and standard deviations (SD) of carcass characteristics, chemical body composition, accretion rates, daily gain, daily feed intake and food conversion ratio measured on pigs of the F_2 _generation. Click here for file

Additional File 2 **Table S2. **Phenotypic correlations between the traits for which QTL effects were detected. Click here for file
