# Supplementary material for: Genomic scan for quantitative trait loci of chemical and physical body composition and deposition on pig chromosome X including the pseudoautosomal region of males
Source: Genet Sel Evol. 2009 Mar 11;41(1):27. doi: 10.1186/1297-9686-41-27 (PMC2666071; doi:10.1186/1297-9686-41-27)
Supplement: Additional File 1 — Table S1. Means and standard deviations (SD) of carcass characteristics, chemical body composition, accretion rates, daily gain, daily feed intake and food conversion ratio measured on pigs of the F2 generation. [file 1297-9686-41-27-S1.doc]

**Table S1 -** Means and standard deviations (SD) of carcass characteristics, chemical body composition, accretion rates, daily gain, daily feed intake and food conversion ratio measured on pigs of the F_2_ generation

| **Trait** | **Mean** | **SD** | **Number of records** | |
| --- | --- | --- | --- | --- |
| *Carcass characteristics – AutoFOM* | | | | |
| AF average fat thickness (mm) | 22.3 | 4.9 | | 313 |
| AF entire shoulder weight (kg) | 6.18 | 0.41 | | 313 |
| AF shoulder lean meat weight (kg) | 4.58 | 0.41 | | 313 |
| AF entire loin weight (kg) | 6.27 | 0.40 | | 313 |
| AF loin lean meat weight (kg) | 3.76 | 0.35 | | 313 |
| AF entire ham weight (kg) | 13.57 | 0.81 | | 313 |
| AF ham lean meat weight (kg) | 9.51 | 1.05 | | 313 |
| AF entire belly weight (kg) | 9.17 | 0.55 | | 313 |
| AF lean content (%) | 50.5 | 6.4 | | 313 |
| AF lean content of belly (%) | 43.7 | 7.9 | | 313 |
| *Dissected carcass cuts* | | | | |
| Entire neck weight (kg) | 5.32 | 0.51 | | 306 |
| Neck weight without external fat (kg) | 4.16 | 0.43 | | 306 |
| External neck fat weight (kg) | 1.16 | 0.29 | | 306 |
| Entire shoulder weight (kg) | 8.45 | 0.56 | | 307 |
| Shoulder weight without external fat (kg) | 5.91 | 0.58 | | 307 |
| External shoulder fat weight (kg) | 1.40 | 0.26 | | 307 |
| Entire loin weight (kg) | 9.16 | 0.73 | | 308 |
| Loin weight without external fat (kg) | 6.65 | 0.62 | | 308 |
| External loin fat weight (kg) | 2.51 | 0.65 | | 308 |
| Entire ham weight (kg) | 16.91 | 1.00 | | 310 |
| Ham weight without external fat (kg) | 11.57 | 1.09 | | 310 |
| External ham fat weight (kg) | 2.57 | 0.49 | | 310 |
| Belly weight (kg) | 6.46 | 0.66 | | 308 |
| Jowl weight (kg) | 1.91 | 0.28 | | 306 |
| Thick rib (kg) | 1.44 | 0.22 | | 307 |
| Flank weight (kg) | 1.79 | 0.41 | | 308 |
| Front hock weight (kg) | 1.14 | 0.19 | | 307 |
| Hind hock weight (kg) | 1.43 | 0.14 | | 310 |
| Tail weight (kg) | 0.43 | 0.13 | | 310 |
| Hind claw (kg) | 0.91 | 0.12 | | 310 |
| *Carcass characteristics – standard performance test* | | | | |
| Carcass length (cm) | 107.9 | 49.3 | | 310 |
| Side-fat thickness^1^ (cm) | 3.85 | 0.88 | | 315 |
| Thinnest fat measure^1^ (cm) | 1.73 | 0.55 | | 314 |
| Loin eye area *M.l.t.l.*^1,2^ (cm^2^) | 54.2 | 6.8 | | 314 |
| Fat area *M.l.t.l.*^1,2^ (cm^2^) | 24.5 | 5.9 | | 314 |
| Fat content of belly (%) | 53.5 | 8.3 | | 306 |
| Fat area of belly (cm^2^) | 23.8 | 6.8 | | 306 |
| *Chemical body composition* | | | | |
| Intramuscular fat content (%) | 1.343 | 0.542 | | 313 |
| Protein content of loin (%) | 24.215 | 2.066 | | 313 |
| Protein content of FFS, 30 kg^3^ (%) | 18.656 | 0.524 | | 299 |
| Protein content of FFS, 60 kg^3^ (%) | 20.115 | 0.419 | | 305 |
| Protein content of FFS, 90 kg^3^ (%) | 21.209 | 0.426 | | 311 |
| Protein content of FFS, 120 kg^3^ (%) | 21.960 | 0.506 | | 302 |
| Protein content of FFS, 140 kg^3^ (%) | 22.359 | 0.543 | | 302 |
| Protein content of empty body, 30 kg^3^ (%) | 16.643 | 0.065 | | 310 |
| Protein content of empty body, 60 kg^3^ (%) | 16.477 | 0.047 | | 305 |
| Protein content of empty body, 90 kg^3^ (%) | 16.359 | 0.045 | | 311 |
| Protein content of empty body, 120 kg^3^ (%) | 16.282 | 0.051 | | 302 |
| Protein content of empty body, 140 kg^3^ (%) | 16.242 | 0.053 | | 302 |
| Lipid content of empty body, 30 kg^3^ (%) | 10.845 | 2.920 | | 310 |
| Lipid content of empty body, 60 kg^3^ (%) | 18.045 | 2.000 | | 305 |
| Lipid content of empty body, 90 kg^3^ (%) | 22.832 | 1.773 | | 311 |
| Lipid content of empty body, 120 kg^3^ (%) | 25.813 | 1.926 | | 302 |
| Lipid content of empty body, 140 kg^3^ (%) | 27.308 | 1.987 | | 302 |
| *Chemical accretion rates* | | | | |
| PAR, 30- 60 kg (kg/day) | 0.110 | 0.018 | | 300 |
| PAR, 60-90 kg (kg/day) | 0.135 | 0.023 | | 300 |
| PAR, 90-120 kg (kg/day) | 0.125 | 0.022 | | 299 |
| PAR, 120-140 kg (kg/day) | 0.115 | 0.031 | | 292 |
| LAR, 30-60 kg (kg/day | 0.168 | 0.040 | | 300 |
| LAR, 60-90 kg (kg/day) | 0.271 | 0.060 | | 300 |
| LAR, 90-120 kg (kg/day) | 0.274 | 0.069 | | 301 |
| LAR, 120-140 kg (kg/day) | 0.267 | 0.099 | | 293 |
| *Daily gain, feed intake and food conversion traits* | | | | |
| DG, 30-60 kg (kg/day) | 0.677 | 0.114 | | 315 |
| DG, 60-90 kg (kg/day) | 0.838 | 0.138 | | 312 |
| DG, 90-120 kg (kg/day) | 0.779 | 0.140 | | 313 |
| DG, 120-140 kg (kg/day) | 0.718 | 0.193 | | 313 |
| DFI 60-90 kg (kg/day) | 2.467 | 0.361 | | 312 |
| DFI 90-120 kg (kg/day) | 2.818 | 0.376 | | 313 |
| DFI 120-140 kg (kg/day) | 2.815 | 0.496 | | 313 |
| FCR 60-90 kg (kg feed/kg gain) | 2.975 | 0.379 | | 312 |
| FCR 90-120 kg (kg feed/kg gain) | 3.678 | 0.517 | | 313 |
| FCR 120-140 kg (kg feed/kg gain) | 4.214 | 1.975 | | 313 |

^1^collected at the 13^th^/14^th^ rib interface; ^2^measured on *musculus longissimus thoracis et lumborum*.

Definition of symbols: FFS, fat free substance; DG, daily gain; PAR, protein accretion rate; LAR, lipid accretion rate; DFI, daily feed intake; FCR, food conversion ratio

^3^The prediction equations for estimating these traits based on total body water determined by deuterium dilution technique and the correlations between predicted and chemically analyzed components are presented in Landgraf *et al.* [15]
