# Supplementary material for: Genomic scan for quantitative trait loci of chemical and physical body composition and deposition on pig chromosome X including the pseudoautosomal region of males
Source: Genet Sel Evol. 2009 Mar 11;41(1):27. doi: 10.1186/1297-9686-41-27 (PMC2666071; doi:10.1186/1297-9686-41-27)
Supplement: Additional File 2 — Table S2. Phenotypic correlations between the traits for which QTL effects were detected. [file 1297-9686-41-27-S2.doc]

**Table S2 -** Phenotypic correlations between the traits for which QTL effects were detected

|  | Loin weight without external fat (kg) | Neck weight without external fat (kg) | Jowl weight (kg) | Protein content of FFS, 30 kg (%) | Lipid content of empty body, 30 kg (%) | Protein content of empty body, 30 kg (%) | DFI 120-140 kg (kg/day) | LAR, 90-120 kg (kg/day) |
| --- | --- | --- | --- | --- | --- | --- | --- | --- |
| Entire loin weight  (kg) | 0.556 | 0.181 | 0.004 | -0.070 | -0.084 | 0.084 | 0.057 | 0.018 |
| Loin weight without external fat (kg) |  | 0.510 | -0.297 | -0.208 | -0.203 | 0.203 | -0.159 | -0.210 |
| Neck weight without external fat (kg) |  |  | -0.139 | -0.160 | -0.156 | 0.156 | -0.141 | -0.088 |
| Jowl weight  (kg) |  |  |  | 0.122 | 0.146 | -0.146 | 0.159 | 0.204 |
| Protein content of FFS, 30 kg (%) |  |  |  |  | 0.996 | -0.995 | -0.007 | 0.124 |
| Lipid content of empty body, 30 kg (%) |  |  |  |  |  | -1.000 | 0.033 | 0.126 |
| Protein content of empty body, 30 kg (%) |  |  |  |  |  |  | -0.033 | -0.127 |
| DFI 120-140 kg (kg/day) |  |  |  |  |  |  |  | 0.333 |

Definition of symbols: FFS, fat free substance; DFI, daily feed intake; LAR, lipid accretion rate
